# Re-updating the taxonomy of *Kluyvera* genus for a better understanding of CTX-M β-lactamase origin

**DOI:** 10.1128/spectrum.04054-23

**Published:** 2024-09-25

**Authors:** Maria Margarita Rodriguez, Gabriel Gutkind

**Affiliations:** 1 Universidad de Buenos Aires, Facultad de Farmacia y Bioquímica, Instituto de Investigaciones en Bacteriología y Virología Molecular (IBaViM), Buenos Aires, Argentina; 2 Consejo Nacional de Investigaciones Científicas y Técnicas (CONICET), Buenos Aires, Argentina; Universidade de Sao PauloMicrobiology, São Paulo, Brazil

**Keywords:** ESBL, *K. ascorbata*, *K. cryocrescens*, *K. intermedia*, *Kluyvera genomo*sp. 5, *Kluyvera genomo*sp. 6

## Abstract

The taxonomic definitions within the *Kluyvera* genus are still unclear, as several deposits might belong to misidentified species or genus or genome assemblies comprehend large indeterminate nucleotide zones. In this study, we performed a comparative phylogenomic analysis of *Kluyvera* chromosomes and other selected *Enterobacterales*. We also included the genomic analysis of chromosomal *bla*_CTX-M/KLU_ from *Kluyvera* isolates and assigned the plasmid-encoded *bla*_CTX/M_ genes. The study allowed us to propose a new *Kluyvera* genomospecies and to better define *Kluyvera* genomosp. 5. Two new CTX-M sub-groups could also be suggested. Even if no chromosomal *bla*_CTX-M/KLU_ gene can be found in *K. intermedia* and *Kluyvera* genomosp. 6, accurate identification can be achieved by using these gene sequences in the remaining strains.

**IMPORTANCE:**

The use of whole-genome sequencing (WGS) accelerated the identification of new *Kluyvera* species proposals, but a rigorous analysis of these sequences is needed for a better definition, including preexisting, and even established species. *Kluyvera* genomosp. 5 could be more clearly defined, and, among isolates that do not produce a chromosome-encoded CTX-M enzyme, true *K. intermedia* should be kept within the genus as well as a new genomospecies (*Kluyvera* genomosp. 6) different from *K. intermedia*. We could clean up true *Kluyvera* from those that deserved transfer to other genera, and some deposits as *K. ascorbata*, *K. cryocrescens*, *K. georgiana*, and several *Kluyvera* sp. to the real species. Two new sub-groups of CTX-M enzymes could be proposed. The accurate identification of the chromosome-encoded *bla*_CTX-M/KLU_ gene in *Kluyvera* isolates could be a useful taxonomic tool to guide the species classification.

## INTRODUCTION

CTX-M enzymes represent the most prevalent group of extended-spectrum β-lactamases (ESBLs) among pathogens around the world and are considered a global pandemic (1–3). There are around 265 variants of the CTX-M, including both plasmid- and chromosome-encoded variants in *Kluyvera* species (β-Lactamase DataBase: http://bldb.eu/) (4), distributed in at least six gene clusters differing in less than 5% amino acid sequence within each group, as previously reported.

Starting in 2001, sequencing of different *Kluyvera* isolates allowed to propose their chromosomal *bla*_CTX-M/KLU_ counterparts as progenitors of the clinically relevant plasmidic variants (5–7), and by 2004, it was already known that some of them were not progenitors but direct counterparts that could be directly recruited by plasmid platforms that allow their expression as ESBLs (8, 9). Since then, several other plasmidic counterparts, with assigned alleles, were found in different *Kluyvera* isolates, as already reviewed.

However, the information on the origin of the CTX-M family is still obscure. For example, the same species have been proposed as the origin for different subgroups or different species as the origin of a single subgroup, based on what could be considered erroneous classifications according to current whole-genome sequence (WGS) data, including data deposited under incorrect species designation. This could lead to a more complex scenario considering some recent taxonomic proposals. Sequence information for assigning a species designation in analyzed isolates moved from 16S rDNA to the use of some genes (or concatenated genes) as markers, and today, the availability of more WGS data may allow for a more discriminative and, supposedly, stable taxonomic grouping, but only if each deposit is verified for the quality of the information.

The use of WGS crude data accelerated the appearance of new species proposals (10), or a better definition of preexisting ones, and also entailed the availability of a higher number of sequences within *Kluyvera* but resulted in identification indeterminations.

The genus *Kluyvera* is widely distributed in diverse niches. Members of this genus have been isolated from water, sewage, food, soil, animals, human clinical specimens, and the environment. Although *Kluyvera* has been sporadically reported as the cause of clinically relevant diseases until a decade ago, recent studies reveal an increasing number of reports on clinical isolates. The ability to act as an opportunistic pathogen is, perhaps, underestimated due to already known identification difficulties (11–14). Differentially to older reports, *Kluyvera* are now also hosts for mobile genetic platforms harboring different β-lactamase-encoding genes other than *bla*_CTX-M_ (e.g., TEM, serine-β-lactamases, and metallo-β-lactamases) (15–19).

Chromosomal counterparts belonging to the five original sub-groups of acquired CTX-M β-lactamases have been found in *Kluyvera* (1), and three more were added more recently (11). Thus, even if KLUC-1 (from *Kluyvera cryocrescens*) was originally proposed as an ancestor for the CTX-M-1 cluster, now *Kluyvera* genomosp. 5 producing CTX-M-37, -10, -246, or related group 1 enzymes are the most likely origin, while KLUC enzymes are an independent sub-group of β-lactamases (11). KLUA (from *Kluyvera ascorbata*) remains as ancestor for the CTX-M-2 cluster. The CTX-M-8 sub-group was recently reported as derived from a chromosomal counterpart in *Kluyvera* genomosp. 3 (11). Chromosome-encoded KLUY-1–4 from *Kluyvera georgiana* were proposed for the CTX-M-9 sub-group and CTX-M-213 from the chromosomal counterpart *Kluyvera* genomosp. 2. CTX-M-78, a chromosome-encoded β-lactamase from *Kluyvera georgiana* 14751, is related to the CTX-M-25 sub-group and is still considered as a probable origin of this cluster. Even if *Kluyvera sichuanensis* 13608 chromosomal β-lactamase is related to the CTX-M-2 sub-group (the encoded β-lactamase exhibits 86% nucleotide identity and 91% amino acid identity with CTX-M-2), it has been already proposed as a new sub-group. *Kluyvera* genomosp. 1 strain L2 β-lactamase also represents a new sub-group of CTX-Ms (11).

In this study, we performed a comparative phylogenomic analysis of *Kluyvera* isolates chromosome and other selected genera of the order *Enterobacterales* (20) to group *Kluyvera* isolates that belong to the same clade, re-classifying the outsiders. We included the genomic analysis of the whole *bla*_CTX-M_ sequences available and the chromosomal genes present in all *Kluyvera* species, considering that these latter genes may be useful for a better taxonomic assignment.

## MATERIALS AND METHODS

### Genomic DNA analysis

Chromosomes from 76 *Kluyvera* spp. isolates and 74 from different genus of *Enterobacterales* (*Phytobacter*, *Metakosakonia*, *Kosakonia*, *Yokenella*, *Trabulsiella*, *Pluralibacter*, *Raoultella*, *Erwinia*, *Hafnia*, *Leclercia*, *Lelliottia*, *Edwardsiella*, *Chania*, *Yersinia*, *Serratia*, *Siccibacter*, *Shimwellia,* and *Klebsiella*) were downloaded from the NCBI database (https://www.ncbi.nlm.nih.gov/genome/) between November 2022 and June 2023 (≤250 contigs). The strains are listed in the supplemental material (see Table S1). This selection of *Enterobacterales* was performed following the results of the article by Alnajar and Gupta (20), in which several conserved proteins were described as taxonomically useful to separate *Kluyvera* species and other *Enterobacteriaceae* (*Klebsiella* spp., *Raoultella* spp., *Trabulsiella* spp., *Yokenella* spp., *Hafnia* spp., *Erwinia* spp., *Leclercia* spp., and *Lelliotta* spp.) within the *Klebsiella* clade. *Kosakonia* sp. and *Phytobacter* sp. were included due to previous average nucleotide identity (ANI) results with some of the isolates received as *Kluyvera* (11).

Chromosomal assembly or SRA sequences (https://www.ncbi.nlm.nih.gov/sra/) were analyzed using Unicycler Version 0.5.0 (21) for *de novo* assembly paired forward and reverse reads, resulting in fasta files. Chromosomal assembly data from isolates were used to perform ANI using the OrthoANIu (https://www.ezbiocloud.net/tools/ani) (22), confronting each genome with the corresponding representative genome (Table S2). Minimal cutoff points of 95% OrthoANI values were considered to represent species delineation.

### Genome phylogeny

We performed all phylogenomic analyses in Galaxy Version 3.13.0 + galaxy s2 platform. Prokka (v. 1.12) was used to produce “*.gff3” output files for each strain (23), and Roary pan-genome pipeline Version 3.13.0 was used for genome alignment (24). All alignments were used as entries in an single nucleotide polimorfisms (SNP)-distance analysis, obtaining the SNP-distance matrix (25); this matrix was displayed only for *Kluyvera* isolates as a seaborn heatmap obtained with Python (v. 3.13.114.0; https://www.python.org/) (Fig. S1). The SNP values were analyzed by Gubbins to reorganize the core genome by removing the recombination in genomes (26). The analysis of the *bla* genes was conducted using ClustalX (http://clustalx.software.informer.com/2.1/) to align all sequences. The molecular evolution model was estimated with IQ-Tree (Version 2.1.2) (27). The phylogenetic trees were built with PhyML (v.3.1) using the maximum-likelihood method, with 1,000 bootstraps replications using ultrafast bootstrap (28, 29). The resulting phylogenetic trees were visualized and edited using the FigTree program (http://tree.bio.ed.ac.uk/software/figtree/). All phylogenetic trees were midpoint rooted.

## RESULTS

### Genome analysis

**FIG 1:**
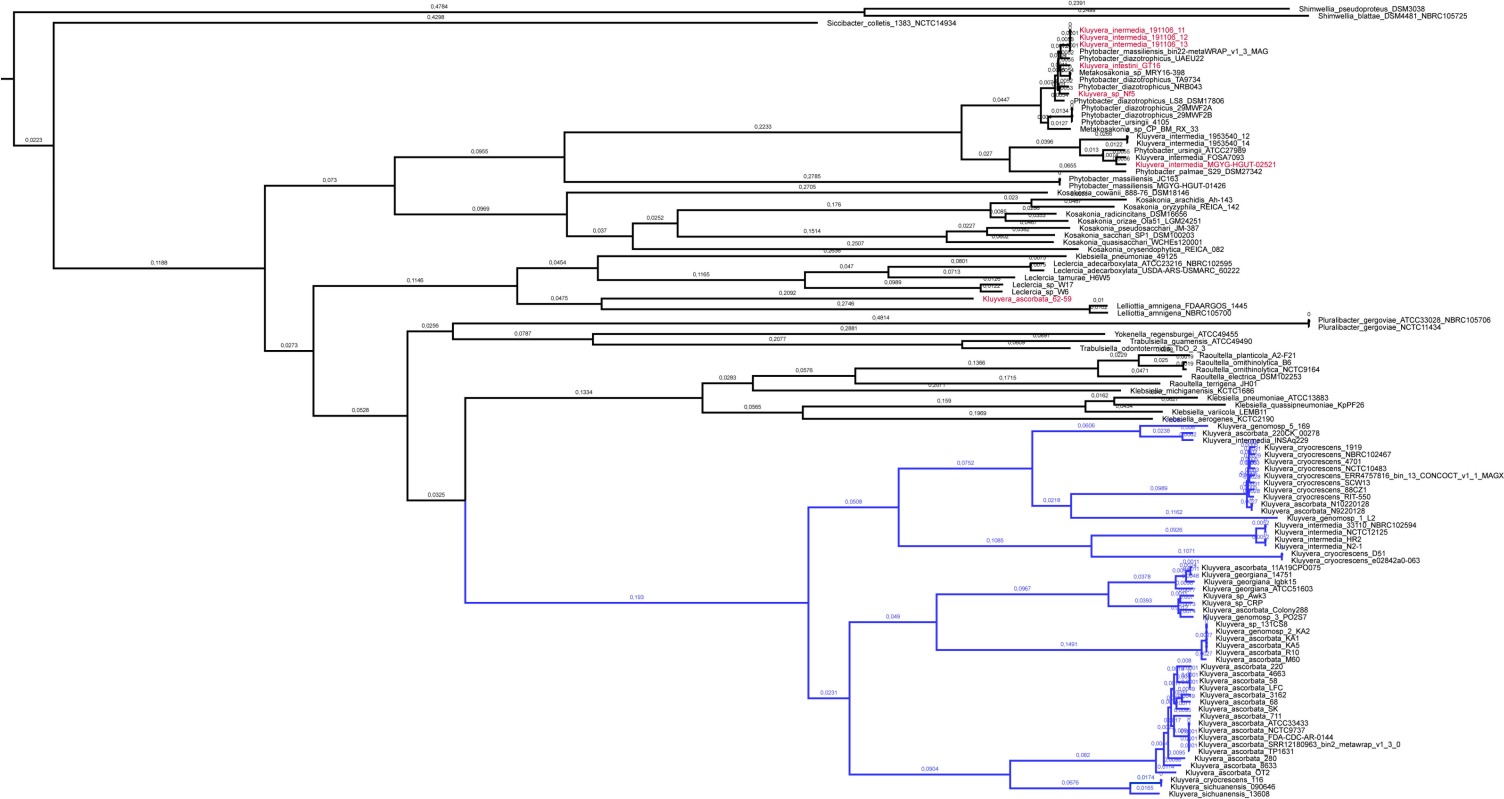
Phylogenetic tree of the chromosomes from *Kluyvera* and other related *Enterobacterales* was performed with IQ-Tree using the maximum-likelihood method based on 67,985 core gene SNPs (bootstrap replications: 1,000). *Kluyvera* genus clade is colored with blue lines. Red names represent the *K. ascorbata* 62–59 genome, *Kluyvera intestinii* GT16, *Kluyvera* sp. Nf5, and *K. intermedia* isolates grouping with *P. diazotrophicus* and *P. ursingii*.

Figure 1 represents the middle point rooted phylogenetic tree of the included *Enterobacterales* (150 complete chromosomes), including the named *Kluyvera* isolates (Table S1). Most of the *Kluyvera* strains group in the same clade. Nevertheless, *Kluyvera intermedia* 1951106–13, 19511106_12, and 1951106_11 isolates, *Kluyvera intestini* GT16, and *Kluyvera* sp. Nf5 group with *Phytobacter diazotrophicus*. On the other hand, *K. intermedia* MGYG-HGUT-025521, FOSA7093, 1953540_12, and 1953540_14 group with *Phytobacter ursingii* (and should be excluded for any sequence analysis within the genus). *K. ascorbata* 62–59 has as closest relatives several *Enterobacter* species and does not harbor a chromosome-encoded CTX-M variant but a class A serine β-lactamase displaying 85% nucleotide identity with a deposited sequence from *Enterobacter cloacae* isolate (GenBank: CP035633.1, data not shown). This suggests that this isolate may belong to the *Enterobacter* genus and not to *Kluyvera*. Unfortunately, sequence quality reveals some errors, and therefore, sequences from *Enterobacter* species isolates were not included in the phylogenomic analysis. Anyway, this deposit is clearly out of our selected genus.

Similarly, our strain 4105 (received as *Kluyvera* sp.), *Metakosakonia* sp. MRY16-52, and CPBM-RX-33 seem to be wrongly identified, as they group with *P. diazotrophicus*.

**FIG 2:**
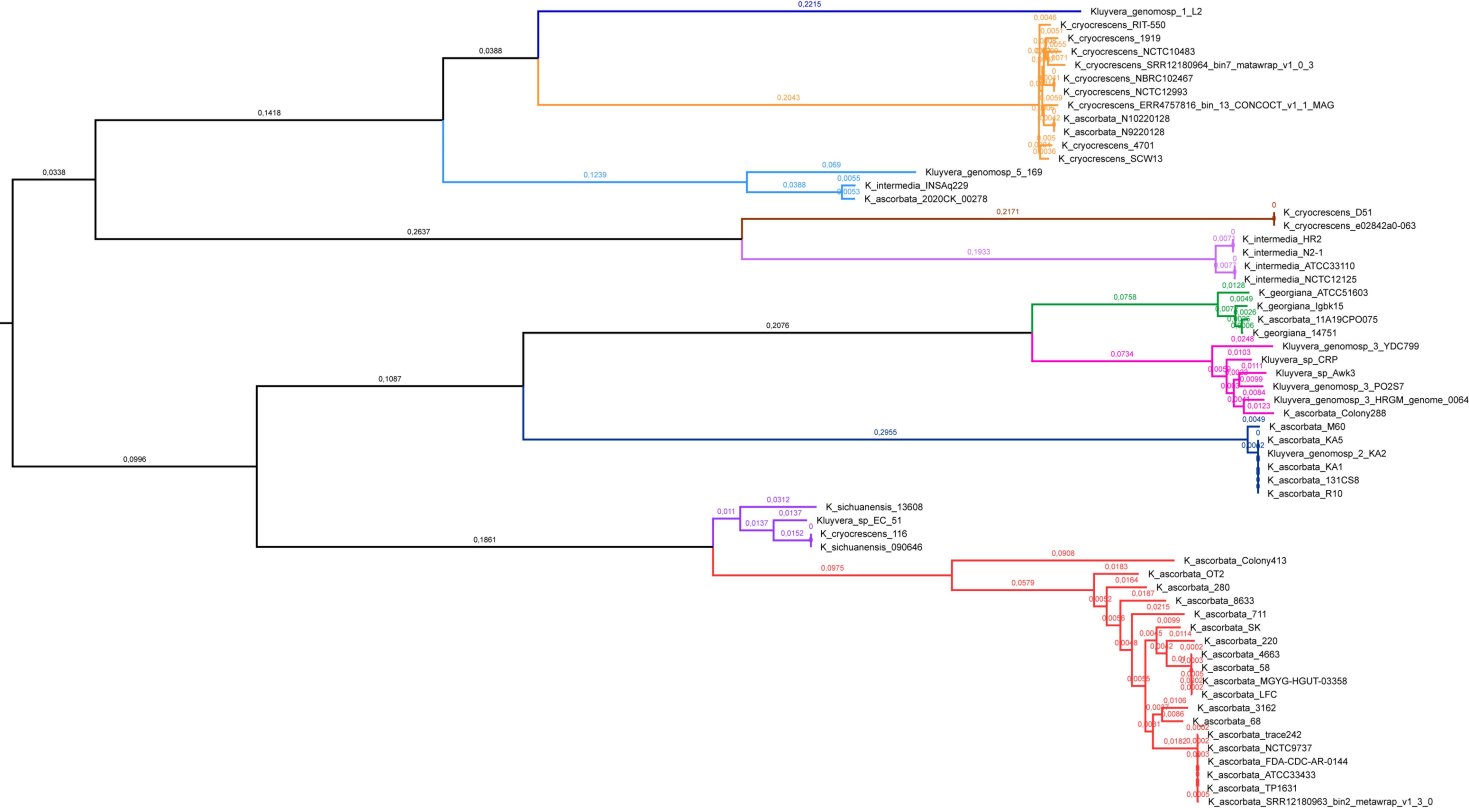
Phylogenetic tree of the chromosomes from *Kluyvera* was obtained with PhyML using the maximum-likelihood method based in 35,579 core gene SNPs (bootstrap replications: 1,000). Color lines represent each clade (group) of isolates classified according to the species suggested (from top to bottom): *Kluyvera* genomosp. 1 (dark blue), *K. cryocrescens* (orange), *Kluyvera* genomosp. 5 (light blue), *Kluyvera* genomosp. 6 (brown), *K. intermedia* (violet), *K. georgiana* (green), *Kluyvera* genomosp. 3 (magenta), *Kluyvera* genomosp. 2 (blue), *K. sichuanensis* (dark purple), and *K. ascorbata* (red).

The rest of *Kluyvera* isolates that group in the same clade are represented in the middle point rooted phylogenetic tree as shown in Fig. 2; Fig. S1; Tables S2 and S3. The *Kluyvera* genus seems to be composed of ten sub-clades including up-to-date 13 misidentified genomes. The range of SNP differences is displayed in parenthesis:

1. *K. georgiana* ATCC51603, 14751, Igbk15, WCH1410, and Colony 392 strains and the misidentified *K. ascorbata* 11A19CP0075 (≤300).
2. *Kluyvera* genomosp. 3 PO2S7, YDC799 strains, two *Kluyvera* spp. Awk3 and CRP, and the misidentified *K. georgiana* Colony288 and *K. georgiana* HRGM_genome_0064 strains (≤300).
3. *Kluyvera* genomosp. 2 KA2 and MGYG-HGUT-02491 and the misidentified *K. ascorbata* KA1, KA5, R10, M60, and 131-CS8 isolates (≤73).
4. *K. ascorbata* isolates including ATCC33433, NCTC9737, 220, 4463, 58, LFK, MGYG-HGUT-03358, 3162, 68, SK, 711, FDA_CDC_AR-0144, SRR12180963, TP1631, Trace242, 280 (ATCC 14236), 8633, OT2, SRR12180963_bin_2_metawrap_v1_3_0_MAG (≤ 373), and the conspicuously distant Colony413 strain (1,100–1,200).
5. *Kluyvera sichuanensis* isolates 090646, 13608, *Kluyvera* sp. EC51, and the misidentified *K. cryocrescens* 116 (≤454).
6. *Kluyvera* genomosp. 5 strain 169 and the misidentified *K. ascorbata* 220CK_00278 and *K. intermedia* INSAq229 strains (108 between the misidentified strains and 1,022–1,033 between them and strain 169).
7. *K. cryocrescens* including isolates of *K. cryocrescens* 1919, 4701, RIT-550, 88CZ1, ERR4757816_bin_13-CONCOCT_v1_1_MAG, NCTC 10483, NBRC 102407, SRR108100009_bin_1_metawrap_v1_3_0_MAG, SCW13, NCTC12993, SRR12180964_bin7_metawrap_v1_3_0_MAG, and the misidentified *K. ascorbata* N10220128 and N9220128 strains (≤159).
8. *Kluyvera* genomosp. 1 represented by L2 alone.
9. *K. intermedia* ATCC 33110, NCTC 12125, HR2, and N2-1 strains (≤129).
10. A new *Kluyvera* genomosp. 6 that includes deposits *K croycrescens* D51 and e02842a0-063 strains (0).

### Phylogeny of *bla*_CTX-M_

**FIG 3:**
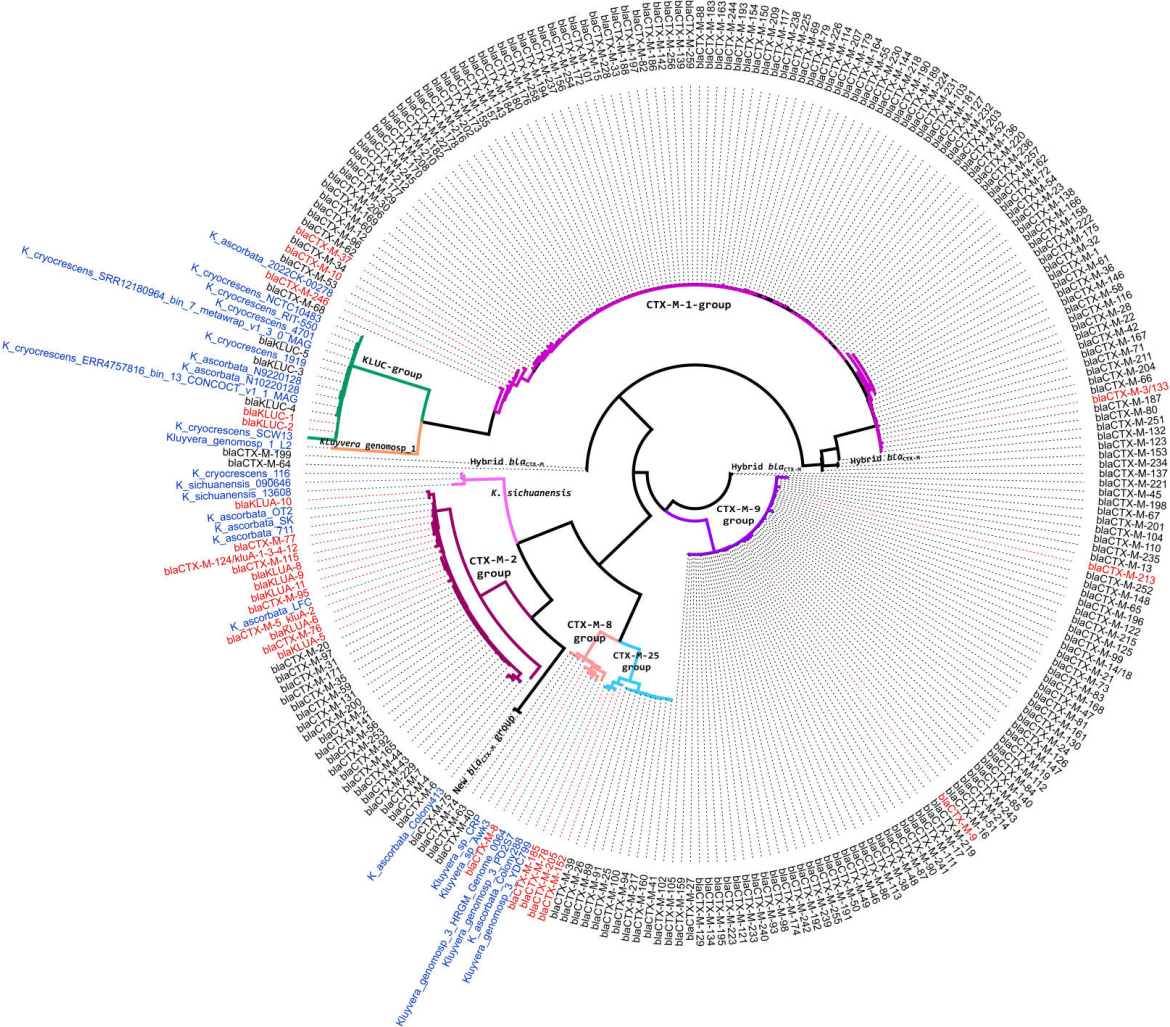
Phylogenetic tree of chromosome and plasmid-borne *bla*_CTX-M/KLU_ genes was obtained with IQ-Tree using the maximum-likelihood method (bootstrap replications: 1,000). Assigned chromosome-encoded *bla*_CTX-M/KLU_ genes are shown in red; names of isolates harboring non-assigned *bla* genes are shown in blue. Each *bla*_CTX-M_ group is represented in a different color.

To complete the analysis, we performed the phylogenetic tree of all *bla*_CTX-M_ genes (http://bldb.eu/), including the assigned and some not yet assigned chromosome-encoded *bla*_CTX-M_ and *bla*_KLU_ from *Kluyvera* isolates (see Fig. 3).

In this phylogenetic tree of all *bla*_CTX-M/KLU_ genes, eight sub-groups of CTX-M-enzymes with at least a chromosomal gene can be evidenced, considering all isolates referred to the original deposit name. The *sub-group* 1 (*bla*_CTX-M-1_ is the representative member) includes a few chromosomal genes, *bla*_CTX-M-3_ from an isolate perhaps erroneously deposited as *K. ascorbata*, and *bla*_CTX-M-37, -10, -146_ from *Kluyvera* genomosp. 5. The *sub-group 2* (*bla*_CTX-M-2_ as the representative member) has all chromosomal genes from *K. ascorbata* like *bla*_KLUA-1_, *bla*_KLUA-3_, *bla*_KLUA-4_, and *bla*_KLUA-12_, which have 100% nucleotide identity with *bla*_CTX-M-124_, *bla*_KLUA-2_ (also deposited as *bla*_CTX-M-5_), *bla*_CTX-M-76_, *bla*_CTX-M-77_, *bla*_CTX-M-95_, and *bla*_CTX-M-115_, and the *bla* gene from Colony413 strain which is the most distant gene with a 94% nucleotide identity with *bla*_CTX-M-2_. The *sub-group 8* (*bla*_CTX-M-8_ as the representative member) includes chromosomal genes from *Kluyvera* genomosp. 3 (YDC799, PO2S7), *Kluyvera* sp. (CRP, Awk3), and the erroneously deposited as *K. ascorbata* (Colony392), related to *bla*_CTX-M-8_ and *bla*_CTX-M-40_. The *sub-group 9* (*bla*_CTX-M-9_ as the representative member) contains the misidentified *K. ascorbata* strain 60, with a chromosomal *bla*_CTX-M-9_ gene, and other *Kluyvera* genomosp. 2 strains (KA2 and the misidentified strains *K. ascorbata* KA1, KA5, R10, and 131-CS8) with a chromosomal *bla*_CTX-M-213_ gene. The *sub-group 25* (*bla*_CTX-M-25_ as the representative member) includes the chromosomal gene *bla*_CTX-M-78_, *bla*_CTX-M-152_, *bla*_CTX-M-185_, and *bla*_CTX-M-205_, all within *K. georgiana*. The *sub-group KLUC* (chromosomal *bla*_KLUC-1_ as the representative member) contains chromosomal *bla*_KLUC_ genes like *bla*_KLUC-2_ (88CZ1 strain), three strains with a single mutation (1919, ERR4757816_bin_13, and SRR12180964_bin_7), two strains with two mutations (N10220128 and N9220128), and two strains with three nucleotide changes (4701 and RIT-550) compared with *bla*_KLUC-5_, named all as *K. cryocrescens* except strains N10220128 and N9220128 erroneously assigned as *K. ascorbata*.

Also, two new sub-groups are described: sub-group “NEW-1,” including only one chromosomal *bla* gene from *Kluyvera* genomosp. 1 strain L2, and sub-group “NEW-2,**”** including the *bla* genes from *K. sichuanensis* strains (090646, 13608) and the erroneously assigned as *K. cryocrescens* 116, all having only 84–86% nucleotide identity to *bla*_CTX-M-76_ gene, their closest relative.

**FIG 4:**
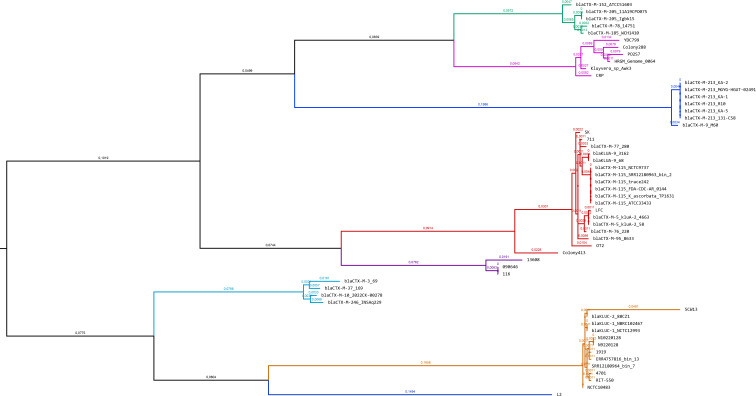
Phylogenetic tree of only chromosome borne *bla*_CTX-M/KLU_ genes from *Kluyvera*, built with IQ-Tree using the maximum-likelihood method (bootstrap replications: 1,000). The lines hold the color pattern of Fig. 2.

“Outlier” plasmid-borne *bla*_CTX-M_ members include (i) *bla*_CTX-M-45_; (ii) a cluster containing the proposed genes encoding for hybrid enzymes derived from CTX-M-1 and CTX-M-9 members like *bla*_CTX-M-64_ and *bla*_CTX-M-199_; (iii) *bla*_CTX-M-132_; (iv) *bla*_CTX-M-123_ and *bla*_CTX-M-153_; (v) *bla*_CTX-M-234_; and (vi) the rest of hybrid *bla*_CTX-M_ genes, resulting in a cluster with *bla*_CTX-M-137_ and *bla*_CTX-M-221_ genes. Figure 4 represents the phylogenetic tree of all chromosomal *bla*_CTX-M/KLU_ genes from *Kluyvera* isolates. The phylogenetic relationship among chromosomal *bla*_CTX-M/KLU_ genes from *Kluyvera* isolates remains conserved when compared to the phylogenetic tree of *Kluyvera* genomes (Fig. 2).

## DISCUSSION

Even if an increase in clinically relevant *Kluyvera* isolates is reported worldwide, only a few studies include genomic data. From them, a vast number of incomplete and/or misassembled sequences are available in databases, leading to misidentifications. In the last few months (August 2023), after we finished our primary analysis, the NCBI database modified the way by which genomic data are shown, announcing some likely errors, i.e., the genome assemblies of *K. intermedia* FOSA7093, *K. georgiana* Colony392, and *K. ascorbata* Colony413 have unusually “too small” lengths, and *Kluyvera* sp. Nf5 isolate “is contaminated” (as examples). This may justify that, upon analysis of the obtained chromosome sequence of *K. georgiana* Colony392, no full chromosome-encoded *bla* gene could be localized, likely due to the presence of a multiple and continuous “N” indetermination zone in the sequence.

1. Most *K. georgiana* isolates and *K. ascorbata* 11A19CP0075, encoding chromosomal enzymes related to sub-group of CTX-M-25, group in the same sub-clade, for which this *K. ascorbata* isolate should be re-classified as *K. georgian*a as well.
2. Confirming genomic clustering most isolates having a chromosomal *bla*_KLUC_ gene belonged to *K. cryocrescens*.
3. Again, all isolates assigned as *Kluyvera* genomosp. 2 displayed a chromosome-encoded from CTX-M-9 sub-group.
4. All the *Kluyvera* isolates harboring a chromosomal *bla*_CTX-M_ related to the plasmidic-encoded *bla*_CTX-M-8_ group in the same sub-clade, belonging to *Kluyvera* genomosp. 3.
5. All chromosome-encoded *bla*_CTX-M/KLU_ genes related to *bla*_CTX-M-2_ are present in the true *K. ascorbata* sub-clade, including the phylogenetically distant Colony413 strain. Regarding *K. ascorbata* Colony413 data, re-sequencing would probably better define if it could be retained in the same cluster (CTX-M-2 sub-group) or if it belongs to a new genomospecies.
6. Other isolates grouped with *K. sichuanensis*. This species represents the origin of a new sub-group of chromosome-encoded CTX-M enzymes (as none of them have been validated number yet, they were designed preliminary as NEW-2 sub-group, to be transferred to the first to be accepted).
7. The chromosome-encoded *bla*_CTX-M-37_ from *Kluyvera* genomosp. 5 strain 169 can be considered the best candidate for sub-group *bla*_CTX-M-1_ origin, and all the different isolates having a chromosomal *bla*_CTX-M-1_ related enzyme should be re-classified as *Kluyvera* genomosp. 5 species as well (11).
8. As previously described, *Kluyvera* genomosp. 1 L2 strain is the single member of this sub-group; it harbors a chromosomal *bla*_CTX-M/KLU_ gene with only 80% nucleotide identity to *bla*_CTX-M-29_ (its closest relative that belongs to the *bla*_CTX-M-1_ sub-group). This isolate would represent the origin of a still not disseminated sub-group of CTX-M enzymes, designed preliminary as NEW-1 sub-group, to be transferred to the first accepted numbered).

As stated before, among isolates that do not display any chromosomal *bla*_CTX-M_ counterparts, some should be retained as *K. intermedia,* while others should be considered as a species different from *K. intermedia*; we propose they should be considered as *Kluyvera* genomosp. 6, to keep the genomospecies numbering correlation. These two species are so far the only ones without a chromosomal *bla*_CTX-M/KLU_ counterpart.

As the genus includes species with or without chromosomal counterparts to the CTX-M family, it can be considered that it might evolve from a common ancestor in which these genes were introduced from an unknown source.

According to our results, several sequences deposited as *K. intermedia*, *Phytobacter massiliensis, Metakosakonia* sp., and *P. ursingii* should be re-classified as *P. diazotrophicus*, and it is likely that other genome deposits as *Phytobacter* or *Metakosakonia* should be also reevaluated. Under a similar approach, other *K. intermedia* should be considered as *P. ursingii*.

A stricter WGS analysis is mandatory to avoid sequence/assembly mistakes that obscure a clear taxonomic evaluation. Even if this is true for any deposit, in the case of microorganisms with few researchers working on them, it is likely that mistakes will remain for longer periods before being amended.

In our study, we observed that (except for the proposed *Kluyvera* species that do not display them) the amplification and sequence analysis of chromosomal *bla*_CTX-M/KLU_ genes can be considered an inexpensive and easy-to-perform taxonomic tool to classify bacterial isolates as members of the genus.

As already mentioned previously, identification within the genus *Kluyvera* is elusive. So far, the newly recognized species cannot be differentiated by conventional biochemical tests or even by MALDI-TOF MS, as they have not been fully incorporated into databases. Moreover, some of the previously defined species are inconsistent with what is inferred by full genome sequencing.

Only genome sequences showing high quality score should be used for the taxonomic classification of potential members of this genus (i.e., FastQC, number of contigs, N50, and coverage). As *Kluyvera* are only infrequent isolates as compared with other *Enterobacterales*, only laboratories with the capacity for sequencing all isolates may benefit from straight sequencing at the clinical laboratory level. Different would be the case for the few research groups working on *Kluyvera* that would be able to make more efficient use of full sequencing, as well as a PCR strategy for identification by using chromosomal *bla*_CTX-M_ sequencing.

Finally, and as already proposed, as at least some plasmid-encoded CTX-M enzymes have a strict identity to chromosome-encoded enzymes (and *vice versa*), different nomenclatures (i.e., KLU or CTX-M) should be avoided, and a single (consensus) nomenclature should be applied univocally for a single enzyme sequence.

## SUPPLEMENTAL MATERIAL

Figure S1 spectrum.04054-23-s0001.jpg Heatmap of SNP distance matrix.

Table S1 spectrum.04054-23-s0002.xlsx Genomes for computational analysis.

Table S2 spectrum.04054-23-s0003.xlsx Proposed genus and species of the *Kluyvera* isolates.

Table S3 spectrum.04054-23-s0004.xlsx SNP distance matrix of *Kluyvera* chromosomes.

ASM does not own the copyrights to Supplemental Material that may be linked to, or accessed through, an article. The authors have granted ASM a non-exclusive, world-wide license to publish the Supplemental Material files. Please contact the corresponding author directly for reuse.
