# Supplementary figures and images for: Re-updating the taxonomy of Kluyvera genus for a better understanding of CTX-M β-lactamase origin
Source: Microbiol Spectr. 2024 Sep 25;12(11):e04054-23. doi: 10.1128/spectrum.04054-23 (PMC11537003; doi:10.1128/spectrum.04054-23)

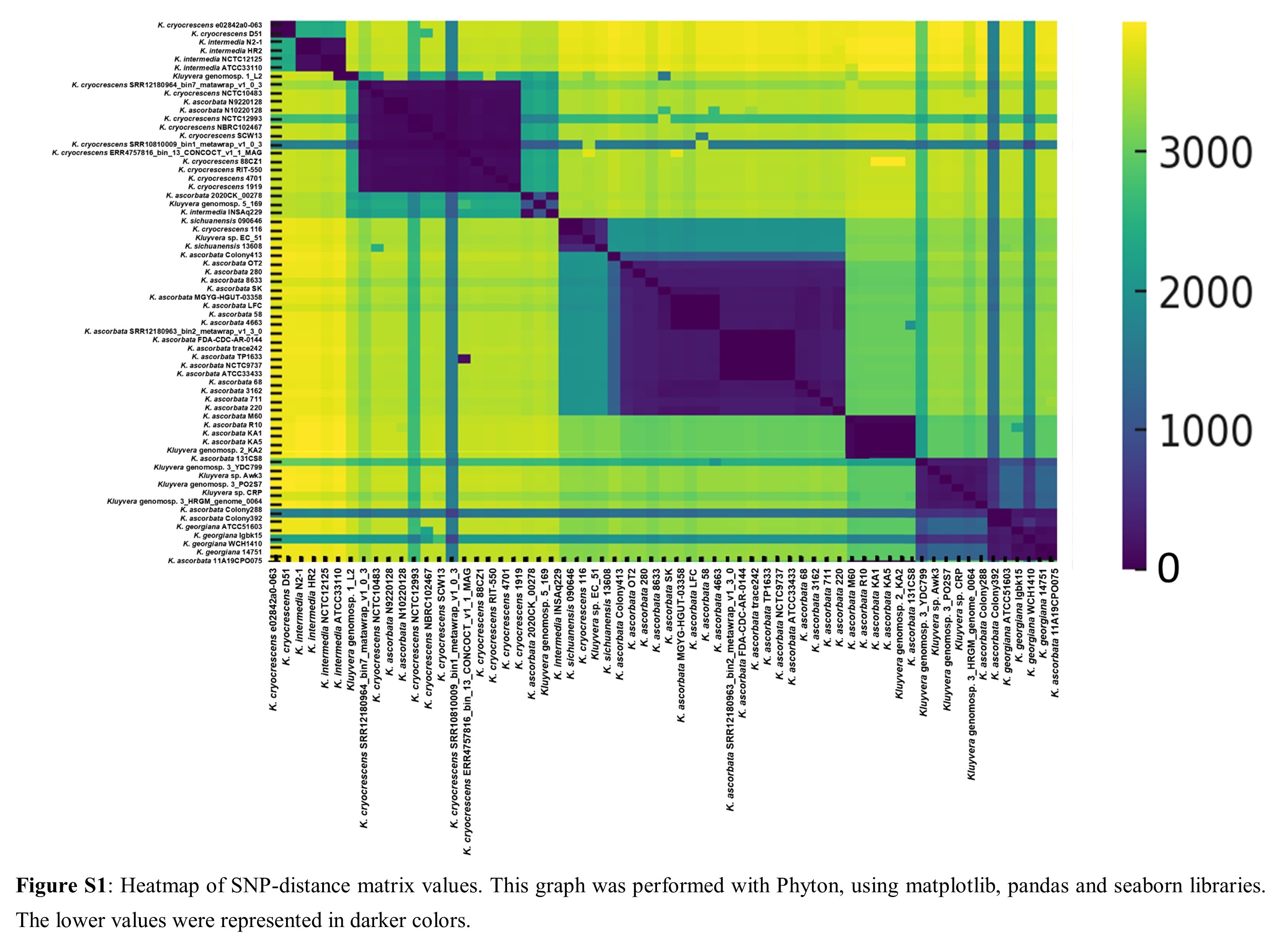

Supplement: Figure S1 — Heatmap of SNP distance matrix. [file spectrum.04054-23-s0001.jpg]
